# Supplementary material for: MicroRNA-deficient mouse embryonic stem cells acquire a functional interferon response
Source: eLife. 2019 Apr 23;8:e44171. doi: 10.7554/eLife.44171 (PMC6529217; doi:10.7554/eLife.44171)
Supplement: Supplementary file 1. [file elife-44171-supp1.docx]

**Table S1. Oligonucleotides used in this study**

|  | **Name** | **Sequence (5’-3’)** |  |
| --- | --- | --- | --- |
| **qRT-PCR** | 7SK forward | GACATCTGTCACCCCATTGA |  |
|  | 7SK reverse | GCCTCATTTGGATGTGTCTG |  |
|  | Actb forward | GGCACCACACCTTCTACAATG |  |
|  | Actb reverse | GGGGTGTTGAAGGTCTCAAAC |  |
|  | Cxcl10 forward | ggtctgagtgggactcaagg |  |
|  | Cxcl10 reverse | gtggcaatgatctcaacacg |  |
|  | Dgcr8 forward | gctgcaggagtaaggacagg |  |
|  | Dgcr8 reverse | tcgagcactgcatactccac |  |
|  | Dgrc8 (exon 3) forward | TCCCAAGAAGAGGCGAATGG |  |
|  | Dgrc8 (exon 3) reverse | CCACGACTTTTGAGCACTGTTT |  |
|  | Dicer forward | ggtcctttctttggactgcca |  |
|  | Dicer reverse | gcgatgaacgtcttccctga |  |
|  | GAPDH forward | TGTGTCCGTCGTGGATCTGA |  |
|  | GAPDH reverse | CCTGCTTCACCACCTTCTTGA |  |
|  | Gata 6 forward | GCAATGCATGCGGTCTCTAC |  |
|  | Gata 6 reverse | CTCTTGGTAGCACCAGCTCA |  |
|  | Gata4 forward | GAAAACGGAAGCCCAAGAACC |  |
|  | Gata4 reverse | TGCTGTGCCCATAGTGAGATGAC |  |
|  | IFNB1 forward | aagagttacactgcctttgccatc |  |
|  | IFNB1 reverse | cactgtctgctggtggagttcatc |  |
|  | Influenza A virus (seg. 5) forward | ATCATGGCGTCTCAAGGCAC |  |
|  | Influenza A virus (seg. 5) reverse | CCGACGGATGCTCTGATTTC |  |
|  | ISG15 forward | aggtctttctgacgcagactg |  |
|  | ISG15 reverse | ggggctttaggccatactcc |  |
|  | MAVS forward | ctgcctcacagctagtgacc |  |
|  | MAVS reverse | ccggcgctggagattattg |  |
|  | MDA5 forward | tcatcgaagcagctgacact |  |
|  | MDA5 reverse | gcctggaacgtagacgacat |  |
|  | Nanog forward | AGGGTCTGCTACTGAGATGCTCTG |  |
|  | Nanog reverse | CAACCACTGGTTTTTCTGCCACCG |  |
|  | Neurog2 forward | GACATTCCCGGACACACAC |  |
|  | Neurog2 reverse | CCAGCAGCATCAGTACCTCC |  |
|  | Oas1 forward | ATGGAGCACGGACTCAGGA |  |
|  | Oas1 reverse | TCACACACGACATTGACGGC |  |
|  | Pou5f1 forward | AGTTGGCGTGGAGACTTTGC |  |
|  | Pou5f1 reverse | CAGGGCTTTCATGTCCTGG |  |
|  | RIG-I forward | cacttcgttcatctctggcg |  |
|  | RIG-I reverse | agagtgaggcagcttccatt |  |
|  | Stat1 forward | TCACAGTGGTTCGAGCTTCAG |  |
|  | Stat1 reverse | GCAAACGAGACATCATAGGCA |  |
|  | TMEV forward | Tgtggacttggacgatgacg |  |
|  | TMEV reverse | cagtatcgcatacgagcggt |  |
|  | TNFα forward | TCCCAGGTTCTCTTCAAGGGA |  |
|  | TNFα reverse | GGTGAGGAGCACGTAGTCGG |  |
| **RT-PCR** | IFNB1 forward | AGCTCCAAGAAAGGACGAACAT |  |
|  | IFNB1 reverse | GCCCTGTAGGTGAGGTTGATCT |  |
| **Cloning** | MAVS pLenti FW EcoRV | GCCGGGATATCATGACATTTGCTGAGGACAAGAC | |
|  | MAVS pLenti RV XbaI | CGGCCCTCTAGATCACTGGGCCAGGCGCCTAC | |
|  | DGCR8 pLenti-FW EcoRV | TCTAGTCCGATATCATGGAGACAGATGAGAGCCC | |
|  | DGCR8 pLenti-RV Xba1 | CTGATCACTCTAGATCACACGTCCACGGTGCACAG | |
| **3’UTR** | RIG-I_3'UTR_FW_XhoI | GCGCGCGCTCGAGCCTCAGGCTTCTCCGTCTCGTG | |
|  | RIG-I_3'UTR_RV_NotI | CGGCCCGCGGCCGCAAATACATAAATTTTTAATTAATT | |
|  | MDA5_3'UTR_FW_XhoI | GCGCGCGCTCGAGCACTTGATTCATGATTATTTTA | |
|  | MDA5_3'UTR_RV_NotI | CGGCCCGCGGCCGCAATAGTATCTGAAATATACATC | |
|  | MAVS_3UTR_FW_XhoI | gcgcgcgctcgagagcctcagctgtatgctgttctc | |
|  | MAVS_3UTR_RV_NotI | cggcccgcggccgctttgcacagataaactcttttaattatcctg | |
| **Probes** | mmu-miR-130a-3p | CAGTGCAATGTTAAAAGGGCAT CCTGTCTC | |
|  | mmu-miR-293-3p | AGTGCCGCAGAGTTTGTAGTGT CCTGTCTC | |
|  | mmu-miR-294-3p | AAAGTGCTTCCCTTTTGTGTGT CCTGTCTC | |
| **CrRNA target mmu-miR-673** | 1 | CCAGAGAAAATGTTGCTCCGGGG | |
|  | 2 | ACCAGAGCTGTGAGCCCCTCAGG | |
|  | Genomic PCR miR-673 locus F | AGCAGTGATGGGTGTGCTAC | |
|  | Genomic PCR miR-673 locus R | TCCATTTCCCATCCCCTTGC | |
|  |  |  |  |
|  |  |  |  |
